# Binding study advice: effect of raising the standards?

**DOI:** 10.1007/s40037-015-0180-1

**Published:** 2015-05-12

**Authors:** Karen M. Stegers-Jager, Axel P. N. Themmen

**Affiliations:** 1 Institute of Medical Education Research Rotterdam, Erasmus MC, PO Box 2040, Room AE-241, 3000 CA Rotterdam, The Netherlands; 2 Department of Internal Medicine, Erasmus MC, Rotterdam, The Netherlands

With great interest we have read the recent Show and Tell article *The binding study advice in medical education: a 2-year experience* by Eijsvogels et al. [1]. Their results largely confirm our own previous findings that the implementation of the binding study advice (or academic dismissal policy as we called it) did not lead to earlier dropout, higher completion rates or an improved study rate during the first 2 years at our medical school [2]. To explain the limited impact of the binding study advice on the study outcomes in medical faculties, Eijsvogels et al. propose a ‘ceiling effect’ rather than a ‘threshold effect’: it is not so much that students reduce their study efforts after having obtained enough ECTS credits, but due to the already high performance level of medical students there is little room for improvement.

Although this explanation sounds plausible, we are not sure whether we totally agree. The reason for this is that we wonder what the effect would be of raising the standards. We believe that in particular non-struggling students may be expected to demonstrate the ability to improve their study progress if they are encouraged to do so. Indeed, Eijsvogels et al. find that more students obtained access to the second year of the study programme after the implementation of the binding study advice.

The ultimate example of standard raising is to set the minimum equal to the maximum, or–put differently–to expect students to obtain all 60 first-year ECTS credits within one year. A couple of years ago this so-called *nominal = normal* policy was implemented at the Erasmus University Rotterdam and more recently (September 2014) also at the Erasmus MC Medical School. A prerequisite for introducing such a system is that medical schools encourage and facilitate students to meet the standard by providing feasible study programmes, student-centred learning and timely feedback on examinations, and allow compensation between exam scores.

Although the effect of the *nominal = normal* policy on study outcomes of our medical students is not yet known, it provides a strong incentive to students whose study progress is not optimal to obtain all first-year credits within one year. Ideally, it becomes just natural to have an optimal study rate and focusing on the minimum standards required becomes pointless.
